# Jesting

**Published:** 1878-12

**Authors:** 


### JESTINGS.

Sometimes a narrator of the ludicrous may make a mistake and cause a laugh on the "wrong side of the mouth," and what at one time may be excessively amusing, may at another fail to raise even a smile, for a great deal depends on the state of the mind, but there is scarcely one in a thousand who would not appreciate some one of the following:

"Out of sight, out of mind," but we don't see it; we lost our wallet the other day and it hasn't been out of our mind since.

OLD AND NEW.—"What do Arabs of the desert live on, pa?" asked a roguish girl of her father. "Fudge, Nelly, that's an old conundrum. They live on the *sand which is* (sandwiches) there." "Yes, but, pa, how do they get 'em?" "Well, really, Nelly, you have me there. I give it up." "Why, pa, you know that the sons of *Ham are bred and mustered* in the wilderness?" "Come, come, my daughter, that is too killing; don't say another word." "Oh, yes, do tell me what they eat on their sandwiches." "Eat on 'em; why what do they eat on 'em!" "Butter, to be sure." "Butter? How do they get their butter?" "Why, you know, pa, that when Lot's wife was turned into a pillar of salt, all the family *but her* ran into the wilderness."

—"I will bet you a bottle of wine that you will descend from that chair before I ask you twice." "Done," said the gentleman, who seemed determined not to obey the summons so obediently. "Come down." "I will not." "Then stop till I tell you a second time."

—"What's that ar a picture on?" asked a countryman in a print store the other day of the proprietor, who was turning some engravings. "That, sir, is Joshua commanding the sun to stand still." "Du tell! Which is Josh and which is his son?"

—What is that which is always invisible and never out of sight? The letter I.

—Artemus Ward thus describes his perils at sea: "Deth stared us into the face. But we had rather the advantage of Deth. While Deth stared us into the face, thar was about seventy of us starein Deth into the face. The prospect wasn't pleasin' to us. Not much. I don't know how Deth liked it."

—A lady fixed the following letters in the bottom of a flour barrel, and asked her husband to read them: O-I-C-U-R-M-T.

—Mr. Justice Page was renowned for his ferocity upon the bench. While going the circuit, a facetious lawyer named Crowe was asked if "the Judge was not just behind?" "I don't know," said Crowe, "but if he is, I am sure he was never just before."

—Be temperate in diet. Our first parents ate themselves out of house and home.

—A fashionable but ignorant lady, desirous of purchasing a watch, was shown a very beautiful one, the shop-keeper remarking that it went thirty-six hours: "What, in one day?" she asked.

—"Will you please to help me to some of that butter," said a boarder to another. "It is strong enough to help itself."

—"Ma, is the portrait of father torn?" asked a little cherub of three summers. "No, child, why do you ask?" Why, this morning he said, "Darn my picture!"

—"Many men of many minds," is no longer a truth. A gentleman asked a crowd to imbibe, the other day, with one consent they did.

—A little girl on the cars was asked what motive was taking her to the city. "I believe they call it a locomotive," said the little innocent.

—John Randolph is said, upon one occasion, to have visited a race course near the city of New-York. A flash-looking stranger offered to bet him $500 on the result of the race, and, introducing his companion, said: "Mr. Randolph, my friend here, Squire Tompkins, will hold the stakes." "But, sir, squeaked the orator of Roanoke, "who will hold Squire Tompkins, after I give him my money?"

—A few days since, a gentleman called upon some lady friends, and was shown into the parlor by a servant girl. She asked him what name she should announce, and he, wishing to take them by surprise, replied, "Amicus," (a friend.) The girl seemed at first a little puzzled, but quickly regained her composure, and in the blandest manner possible, observed, "What kind of a cuss did you say, sir?"

—"Can you reach me a potatoe," said a countryman to a gentleman near him at the table, who stretching his fork towards the dish, as if to ascertain the fact, sat down, saying, "Yes, sir," but nary a tater.

—A few weeks after a late marriage, the husband had some peculiar thoughts when putting on his last clean shirt, as he saw no appearance of a "washing." He thereupon rose earlier than usual one morning and kindled a fire. When hanging on the kettle, he made a noise on purpose to arouse his easy wife. She immediately peeped over the blankets, and then exclaimed: "My dear, what are you doing?" He deliberately responded, "I've put on my last clean shirt, and I'm going to wash one now for myself?" "Very well," replied Mrs. Easy, "you had better wash one for me too."

—Mr. Randolph, the celebrated orator and statesman, was lying on a sofa in the parlor of a tavern, waiting for the stage to come to the door. A dandified chap stepping into the room with a whip in his hand, just come from a drive, and standing before the mirror, arranged his hair and collar, quite unconscious of the presence of the gentleman on the sofa. After attitudidizing a while, he turned to go out, when Mr.

Randolph asked him, "Has the stage come?" "Stage, sir! stage" said the fop: "I've nothing to do with it, sir!" "Oh! I beg your pardon," said Randolph, quietly; "I thought you were the driver."

—"I do not give way to puppies," said a man who met Randolph on a narrow foot way; "I do," replied instantly that remarkable man, and, stepping aside, passed on.

—A traveler stopped at an inn to breakfast, and having drank a cup of what was given to him, the servant asked, "What will you take sir, tea or coffee?" That depends upon circumstances," was the reply. "If what you gave me last was tea, I want coffee; if it was coffee, I want tea. I want a change."

—"What do you know of the defendent, Mr. Thompson? Do you consider him a good musician?" "On that point I wish to swear with great care. I do not wish to insinuate that Mr. Vanslopes is not a good musician. Not at all. But I could not help observing (people will observe queer things at times) that after he commenced playing on the clarionet, a saw-filer, who lived next door, left home, and has never yet been heard of."

—A sailor being asked how he liked his bride, replied: "Why, d'ye see I took her for to be only half of me, as the parson says, but dash me if she isn't twice as much as I. I'm only a tar, but she is a Tartar."

—During a recent trial at Auburn, the following occurred to vary the monotony of the proceedings: "Among the witnesses was one, as verdant a specimen of humanity as one would wish to meet with. After a severe cross-examination, the counsel for the Government paused, and then putting on a look of severity, and an ominous shake of the head, exclaimed: "Mr. Witness, has not an effort been made to induce you to tell a different story?" "A different story from what I have told, sir?" "That is what I mean." "Yes, sir; several persons have tried to get me to tell a different story from what I have told, but they couldn't." "Now, sir, upon your oath, I wish to know who those persons are." "Wall, I guess you've tried 'bout as hard as any of them."

